# Research on the distribution, localization, and morphology of fluorides in the cell walls of tea plant leaves

**DOI:** 10.3389/fpls.2025.1539883

**Published:** 2025-03-06

**Authors:** Chunlei Li, Hongmei Xu, Jing Xu, Jinlei Luo, Peizhi Li, Fei Zhao

**Affiliations:** ^1^ Shandong Provincial University Laboratory for Protected Horticulture/College of Agronomy, Weifang University of Science and Technology, Shouguang, China; ^2^ Department of Tourism and Hotel Management, PingDingShan Vocational and Technical College, Pingdingshan, China; ^3^ College of Tea Science, Xinyang Agriculture and Forestry University, Xinyang, China

**Keywords:** *Camellia sinensis* L., fluoride, cell wall modification, enrichment characteristics, chemical forms

## Abstract

Tea plant leaves exhibit fluorine-accumulating properties, and the excessive intake of fluoride (F) via tea consumption may pose health risks to consumers; however, despite the high-F content in tea plant, signs of F toxicity are absent, suggesting the presence of F tolerance mechanisms within tea plant. This study investigated F accumulation in the cell walls and structural composition of cell walls in leaves of two tea plant varieties from tea gardens: *Camellia sinensis* cv. Nongkangzao, a high-F cultivar, and *C. sinensis* cv. Pingyang Tezao, a low-F cultivar. The results indicate that cell walls are the primary site of F accumulation in tea leaves, accounting for greater than 80.8% of total F, primarily in a water-soluble form. Furthermore, the F in tea leaf cell walls is predominantly located within pectin polysaccharides. In the leaves of Nongkangzao and Pingyang Tezao, the F in pectin accounted for 83.2% and 89.6% of cell wall F, respectively. The fluoride in the cell wall components shows a significant correlation with the metal elements Al, Ca, Mn, and K. The cell wall modifications show that fluoride is closely associated with the amino and carboxyl groups in pectin. Thus, this study aimed to provide an in-depth analysis of the role of tea plant leaf cell walls in F accumulation. In summary, we hypothesize that F in tea plant may directly bind to the amino and carboxyl groups in pectin, or it may bind together with metal elements at these sites in pectin, thereby being fixed within the cell wall. This prevents fluoride from further entering the cell interior and mitigates its damaging effects on intracellular structures. This may be a key mechanism underlying the F tolerance in tea plants.

## 1 Introduction

The tea plant is a typical fluorine-accumulating plant, with fluoride (F) concentrations in mature leaves generally ranging from 871 to 1337 mg/kg and exceeding 2000 mg/kg in older leaves (Shu and Zhang, 2003; Wen et al., 2024). The F content in tea leaves accounts for greater than 90% of the total F within a tea plant. F toxicity is commonly associated with interference in various plant metabolic processes. The entrance of excessive F into a plant inhibits root growth in tea plant, leading to lesions at the tips and edges of leaves. In addition, it hinders the synthesis of proteins and ribonucleic acids, damages cell membranes, disrupts chloroplast structures, and affects the activity of several enzymes involved in metabolic processes (Li et al., 2011; Yang et al., 2015; Cai et al., 2016; Pan et al., 2020). Although F is not an essential element for tea plants, they accumulate large amounts of F at levels tens to hundreds of times higher than those found in typical plants. Moreover, no signs of toxicity or adverse symptoms have been observed in tea plant under practical cultivation conditions, suggesting the presence of mechanisms within tea plants for F enrichment and tolerance.

In recent years, analyses of subcellular F contents in tea plant leaves have identified cell walls as primary subcellular components utilized for F storage. Using differential centrifugation techniques, the distribution of F within the subcellular components of the leaves of three tea plant varieties: *C. sinensis* cv. Fuan Dabai, Wuniuzao, and Fuding Dabai were analyzed. The findings revealed a consistent pattern across these varieties, with the F contents ranking as follows: cell wall > nucleus and chloroplast > soluble components containing ribosomes > mitochondria (Li, 2011). Hu et al. (2019) measured the F content in the subcellular fractions of the leaves from three tea plant varieties: *C. sinensis* cv. Fuyun No. 6, Wuniuzao, and Fuding Dabai, and found significantly higher F levels in the cell wall than in the other components. F in the cell wall accounted for 55.45%–80.49% of the total F content. Similar studies analyzing F distributions across tea plant varieties found the highest F concentrations in the cell wall (Cai et al., 2013; Wang, 2014), suggesting a strong association between the cell wall and F. The cell wall is composed of pectin, hemicellulose, cellulose, and other substances. Currently, the specific distribution, morphological forms, and binding mechanisms of F within these cell wall components remain unclear. In this study, two tea plant varieties were utilized, one high-F and one low-F, to analyze F content within cell wall components, examine the forms of F in the cell wall, and explore the impact of cell wall structure on F accumulation. This research elucidates the distribution, forms, and functional groups involved in F binding within cell wall components, providing a theoretical basis for understanding F tolerance mechanisms in tea plant and developing methods to reduce F levels.

## 2 Materials and methods

### 2.1 Plant materials

The tea leaves used in this experiment were collected from tea gardens in Jufeng Town, Rizhao City, Shandong Province, China (35°17′N,119°15′E, altitude 220m), and included *C. sinensis cv*. Nongkangzao (high-F cultivar, 12 years old) and *C. sinensis cv*. Pingyang Tezao (low-F cultivar, 12 years old). Leaves of the same developmental stage (fourth to fifth leaves) were selected for this experiment on May 2017, and the cell wall was extracted for further use.

### 2.2 Preparation of cell walls

The extraction of cell wall materials was carried out according to the procedure described by Li et al. (2012) with modifications. Fresh tea leaves were ground in an ice bath using a pre-chilled mortar and pestle until the cells were ruptured completely (observed under a light microscope). The mixture was then centrifuged at 1000×g for 10 minutes. The residue was washed three times with 10 volumes of pre-cooled 80% ethanol (cooled to −20°C), followed by a single wash with 10 volumes of a mixture of methanol and chloroform (1:1, v:v) (pre-cooled to −20°C) to remove the water-soluble substances. The residue was then washed with 10 volumes of pre-cooled acetone (cooled to −20°C) to remove the lipid-soluble substances. The remaining material comprised the cell wall, which was freeze-dried and stored for future use.

### 2.3 Analysis of F forms in cell walls

Analysis of F forms in cell walls was carried out according to the procedure described by Wu et al. (2002). A mass of 5 g of cell wall powder was added into a 500 mL stopper Erlenmeyer flask, followed by the addition of 200 mL ultrapure water. This mixture was incubated in a water bath at 70°C for 30 min, shaking every 5 min, and centrifuged at 4000×g for 20 min. The extraction procedure was repeated twice. After the second extraction, a small amount of water was added for washing, and the supernatant was combined and increased to a final volume of 500 mL. The supernatant was considered as the water-soluble F component. The remaining precipitate was transferred to a 150 mL stoppered Erlenmeyer flask containing 50 mL NaAc (1 mol·L^−1^, pH 7.0), shaken at 25°C (200 r·min^−1^) for 2 h centrifugation at 4000×g for 20 min, and the appropriate amount of extraction solution was added for washing. The combined extraction liquid was considered as the exchangeable F component. The precipitate was then extracted with 100 mL of hydroxylamine hydrochloride (0.04 mol·L^−1^, pH 2.0) at 25°C with constant shaking (200 r·min^−1^) for 2 h. After centrifuging at 4000×g for 20 min, the extraction solution was added for washing. The combined extraction liquid was considered as the iron–manganese bound F (Fe–Mn combined F) component. Next, 6 mL of 0.02 mol·L^−1^ HNO_3_ and 20 mL of 30% H_2_O_2_ (pH 2.0) were added to the precipitate and mixed well. The mixture was then incubated in a water bath at 83°C ± 3°C for 2.5 h, shaking every 10 min during the process. After cooling to room temperature, 5 mL of ammonium acetate–nitric acid solution (3.2 mol·L^−1^) was added, and the mixture was diluted to 50 mL. After mixing well, the solution was left to stand at room temperature for 2 h and then centrifuged at 4000×g for 20 min. A small amount of ultrapure water was added for washing, and the retained supernatants were combined. The combined supernatant was considered as the organic-bound F component. The remaining residue was dried at 80°C, and the F content was measured using an alkaline fusion method. This residue F was considered as the residual F component.

### 2.4 Separation of cell wall components

An appropriate amount of cell wall material was added into a centrifuge tube, and a 0.5 mol·L^-1^ imidazole solution adjusted to pH 7.0 was added at a 1:20 ratio (material to solution) for the extraction of chelated pectin. This mixture was incubated at 25 °C under continuous shaking at 200 r·min^-1^ for 24 h. Afterward, the mixture was centrifuged at 6000×g for 20 min, and the supernatant was collected. The residual pellet was subjected to three washes with the same 0.5 mol·L^-1^ imidazole solution (pH 7.0) by centrifugation. The supernatants from each wash were pooled and freeze-dried, while the precipitate was discarded. Subsequently, the residual pellet was further extracted to isolate alkali-soluble pectin using a 50 mmol·L^-1^ Na_2_CO_3_ solution containing 20 mmol·L^-1^ CDTA at a 1:20 ratio (material to solution). This extraction was carried out at 25°C under shaking for 24 h. The supernatant was then separated by centrifugation, and the remaining precipitate was washed three times with the Na_2_CO_3_ extraction solution. The supernatants were combined and freeze-dried. For the subsequent hemicellulose extraction, the precipitate was treated with 4 mol·L^-1^ KOH (at a 1:20 ratio of material to solution) under constant shaking at 25°C for 24 h. Then, the mixture was centrifuged, and the supernatant was neutralized using 4 mol·L^-1^ HCl to reach a pH of 5.0, followed by freeze-drying. Finally, the residual material, primarily composed of cellulose, was freeze-dried for further analysis (Yang et al., 2005).

### 2.5 Modification of cell wall components

#### 2.5.1 Methylation of cell wall

A mass of 2.0 g of tea plant leaf cell wall was weighed and placed in a centrifuge tube. A volume of 120 mL of a mixed solution of formic acid and formaldehyde (2:1, v:v) was added to the tube. The centrifuge tube was shaken at 125 r·min^−1^ for 6 h. After the reaction, the tube was centrifuged at 13000×g for 8 min, and the precipitate was washed. Then, the washed precipitate was freeze-dried to obtain the methylated cell wall. The methylated cell wall was stored at 4°C for further use (Xu, 2011).

#### 2.5.2 Esterification of cell wall

A mass of 2.0 g of tea plant leaf cell wall was weighed and placed in a centrifuge tube. Sequentially, 140 mL of anhydrous methanol and 1.2 mL of concentrated HCl were added, adjusting the HCl concentration to 0.1 mmol·L^−1^. The centrifuge tube was shaken at 125 r·min^−1^ for 12 h. Afterward, the tube was centrifuged at 13000×g for 8 min, and the supernatant was discarded. The resulting precipitate was washed thoroughly and freeze-dried. The dried precipitate represents the successfully esterified cell wall, which was stored at 4°C for future use (Xu, 2011).

#### 2.5.3 Pectinase hydrolysis

A mass of 2.0 g of tea plant leaf cell wall was weighed and placed in a centrifuge tube. A volume of 120 mL of 1% pectinase containing 0.1% bovine serum albumin (BSA) was added to the tube. The centrifuge tube was placed in a 30°C water bath for 30 min. Afterward, the tube was centrifuged at 13000×g for 8 min, the supernatant discarded, and the precipitate was washed with deionized water. Finally, the precipitate was freeze-dried and stored at 4°C for future use (Xu, 2011).

#### 2.5.4 Cellulase hydrolysis

A mass of 2.0 g of tea plant leaf cell wall was weighed and placed in a centrifuge tube. A volume of 120 mL of 1% cellulase (containing 0.1% BSA) was added to the tube. The centrifuge tube was heated in a 30°C water bath for 30 min. Afterward, the tube was centrifuged at 13000×g for 8 min, the supernatant discarded, and the precipitate washed with deionized water. The washed precipitate was then freeze-dried and stored at 4°C for future use (Xu, 2011).

### 2.6 F absorption by the cell wall before and after modification

Varying amounts (0.05 g, 0.15 g) of the modified and unmodified cell walls were weighed accurately, and each sample was placed in a conical flask. A volume of 40 mL of a 500 mg/L F solution was added to the flask. Then, the flask was placed on a thermostatic shaker at 25°C and 125 r·min^−1^ for 30 min to allow F adsorption. Afterward, the solution was filtered, and the F concentration in the supernatant was determined using an F ion-selective electrode. The F adsorption capacity was calculated using the following formula: ${\text{Q}}_{e}=\left({\text{C}}_{0}-{\text{C}}_{1}\right)*\text{V}/1000$

In the formula:


$${\text{Q}}_{e}=\text{ adsorption capacity }\left(\text{mg}\right)$$

$${\text{C}}_{0}=\text{ F concentration before adsorption }\left(\text{mg}/\text{L}\right)$$

$${\text{C}}_{1}=\text{ F concentration after adsorption }\left(\text{mg}/\text{L}\right)$$

V = volume of the reaction solution (mL)

### 2.7 Analysis of the F and metal element contents

The F content in the samples was measured using an F ion-selective electrode (9609BNWP, Thermo Fisher, USA) as described by Hu et al. (2019). According to the Chinese National Standard (GB5009.268-2016), the samples were diluted to the standard volume with deionized water, and the contents of Al, Ca, Cu, Fe, K, Mg, and Mn in the leaf cell walls were quantitatively analyzed using an inductively coupled plasma optical emission spectrometer (ICP-OES 6300, Thermo Fisher, USA).

### 2.8 Data analysis

Microsoft Excel 2010 was used for data analysis and graph production, and SAS software was used for the LSD test (LSD method, *p* < 0.05).

## 3 Results

### 3.1 Proportion of F mass in cell walls relative to fresh leaf F mass

As shown in **Table 1**, a total of 690.0 g of fresh Nongkangzao leaves yielded 136.1 g of dried cell wall material, containing 1162.8 mg of F in the fresh leaves. The F content in the extracted cell wall was 968.7 mg, meaning that the F mass in the cell wall constituted 83.3% of the total F mass in the fresh leaves. Similarly, from 530.0 g of fresh Pingyang Tezao leaves, 88.0 g of dried cell wall material was extracted, with the fresh leaves containing 247.2 mg of F. The F content in the extracted cell wall amounted to 199.8 mg, accounting for 80.8% of the total F mass in the fresh leaves. In both the high-F cultivar, Nongkangzao, and the low-F cultivar, Pingyang Tezao, the F masses in the cell walls represented over 80.8% of the total F mass in the fresh leaves; therefore, F is predominantly concentrated in the cell walls of tea plant leaves.

**Table 1:** Proportion of F mass in the cell wall relative to total F mass in the leaves.

Cultivars	F content (mg/kg)	sample quality (g)	Quality of fresh leaf dry matter (%)	F quality(mg)	Rate of F in cell wall to F in fresh leaves (%)
Fresh leaves of NKZ	4537.3 ± 56.2b	690.0	62.9	1162.8 ± 18.5a	83.3
Cell wall of NKZ	7117.3 ± 103.6a	136.1		968.7 ± 13.6b	
Fresh leaves of PYTZ	1427.5 ± 20.7b	530.0	67.3	247.2 ± 10.8a	80.8
Cell wall of PYTZ	2270.4 ± 28.1a	88.0		199.8 ± 11.2b	

Different lowercase letters indicated significant differences between the same cultivar (*p*<0.05.).

*C. sinensis* cv. Nongkangzao: NKZ, *C. sinensis* cv. PingyangTezao: PYTZ, Same below.

### 3.2 Forms of F in the cell wall

**Figure 1:**
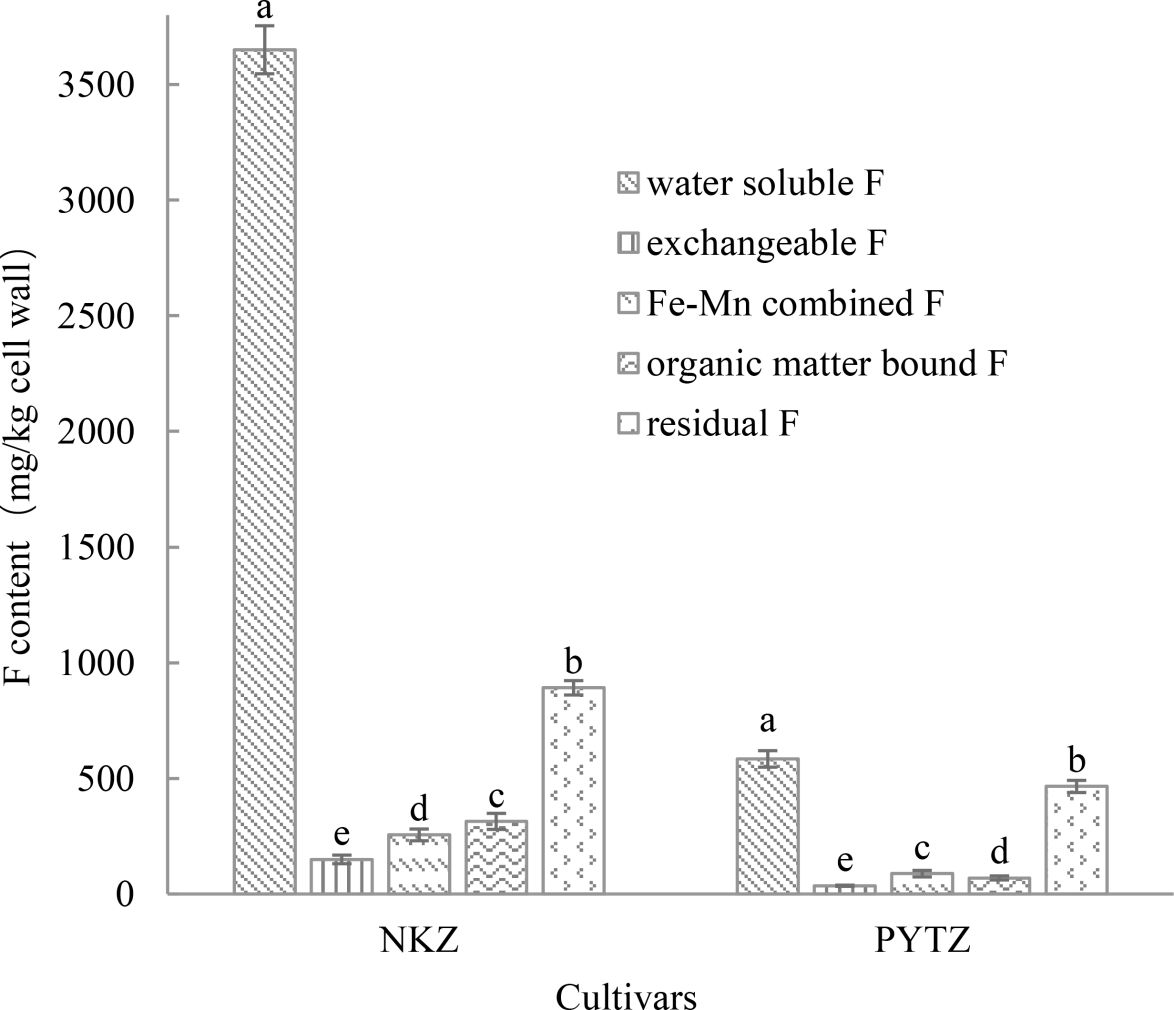
F content in different forms in the tea plant leaf cell wall (mg/kg cell wall). Different lowercase letters in the same cultivar indicate significant differences at *p*<0.05.

The F in the leaf cell walls exists in various forms, including water-soluble F, exchangeable F, Fe–Mn combined F, organic matter-bound F, and residual F. As shown in **Figure 1**, the F in the cell walls of Nongkangzao is primarily in the water-soluble form, with a content of 3650.2 mg/kg, accounting for 68.4% of the total F in the cell wall. The second most abundant form is residual F, accounting for 16.7% of the total F content. Exchangeable F, Fe–Mn combined F, and organic matter-bound F contribute 2.8%, 4.8%, and 5.9%, respectively, to the total F content in cell walls. In the cell walls of Pingyang Tezao, water-soluble F predominates, with a content of 583.9 mg/kg, representing 42.5% of the total F in the cell wall. The next largest proportion is residual F, constituting 33.8%. Exchangeable F, Fe–Mn combined F, and organic matter-bound F represent 2.6%, 6.4%, and 5.1%, respectively, of the total F content in the cell wall. Both tea varieties have a predominant amount of water-soluble F in their cell walls, followed by residual F.

### 3.3 Distribution of F and metal elements in cell wall components

**Figure 2:**
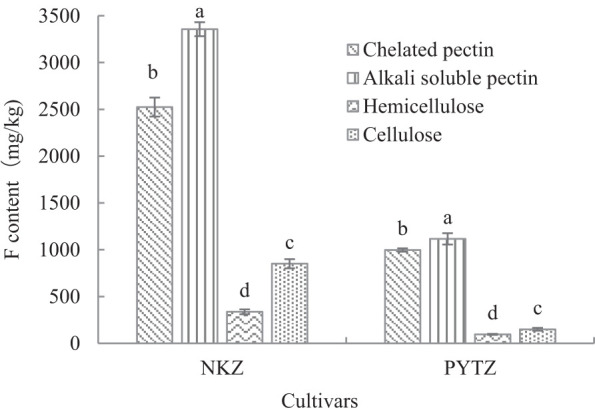
Analysis of F content in cell wall components (mg/kg). Different lowercase letters in the same cultivar indicate significant differences at *p*<0.05.

The cell wall components were separated to investigate the distribution of F elements within each component (**Figure 2**). The results indicated that in the cell wall of Nongkangzao, the F content in pectin accounted for 83.2% of the total F in the cell wall, with chelated pectin contributing 35.7% and alkali-soluble pectin contributing 47.5%. In Pingyang Tezao, the F in pectin accounted for 89.6% of the total F in the cell wall, with chelated pectin contributing 42.3% and alkali-soluble pectin contributing 47.3%. Both varieties showed very low-F contents in hemicellulose, accounting for only 4.8% and 4.1%, respectively, and in cellulose, the F contents were 12.0% and 6.3%, respectively. These findings suggest that F is predominantly concentrated in the pectin component of the cell wall, highlighting the close relationship between cell wall pectin and F.

Further correlation analysis of F and metal elements in the cell wall and its components revealed significant positive correlations between F and Al, Ca, and Mn (*p*<0.05), with correlation coefficients of 0.7396, 0.7252, and 0.7853, respectively. Additionally, F showed a significant negative correlation with K (*p*<0.05), with a correlation coefficient of −0.7269. These results suggest a close association between F and metal elements in the cell wall and its components.

### 3.4 Changes in F content before and after cell wall modification

After modification of the cell wall via methylation, the abundant amino groups in the tea plant leaf cell walls were methylated and subsequently disappeared. The results in **Table 2** showed that methylation modifications significantly reduced the F content in the cell walls of Nongkangzao and Pingyang Tezao. In Nongkangzao, the total F content in the cell wall decreased from 7117.3 mg/kg to 1884.1 mg/kg, representing an F loss of 73.5%. In the control group, the F content decreased to 4625.7 mg/kg, with an F loss of 35.0%. The disappearance of amino groups due to methylation resulted in an F loss of 38.5% in Nongkangzao. In Pingyang Tezao, the total F content in the cell wall decreased from 2270.4 mg/kg to 269.2 mg/kg, representing an F loss of 88.1%, while the control group showed a decrease to 936.8 mg/kg, with an F loss of 58.7%. The disappearance of amino groups due to methylation led to an F loss of 29.4% in Pingyang Tezao. These results indicate that the disappearance of amino groups caused reductions of 29.4%–38.5% in the F content in the cell walls bound to amino groups; therefore, 29.4%–38.5% of the F in the cell wall is closely associated with the amino groups, and these groups play a crucial role in binding F to the cell wall.

**Table 2:** F Loss in cell wall induced by methylation modification.

Cultivars	Treatments	F content (mg/kg)	F loss amount (%)	F loss caused by methylation modification (%)
NKZ	Cell wall	7117.3 ± 101.4a		38.5
	Methylation	1884.1 ± 78.0c	73.5	
	CK	4625.7 ± 51.2b	35.0	
PYTZ	Cell wall	2270.4 ± 70.4a		29.4
	Methylation	269.2 ± 16.9c	88.1	
	CK	936.8 ± 30.6b	58.7	

Different lowercase letters indicated significant differences between the same cultivar (*p*<0.05.).

After modification of the cell wall via esterification, the carboxyl groups in the leaf cell walls were esterified, leading to their disappearance. The results in **Table 3** showed that esterification modification significantly reduced the F content in the cell walls of Nongkangzao and Pingyang Tezao. In Nongkangzao, the total F content in the leaf cell wall decreased from 7117.3 mg/kg to 1713.6 mg/kg, representing an F loss of 75.9%. In the control group, the F content decreased to 4559.0 mg/kg, with an F loss of 35.9%. The esterification of the cell wall resulted in an F loss of 40.0% in Nongkangzao. In Pingyang Tezao, the total F content in the leaf cell wall decreased from 2270.4 mg/kg to 268.4 mg/kg, representing an F loss of 88.2%, while the control group showed a decrease to 802.7 mg/kg, with an F loss of 64.6%. The esterification of the cell wall led to an F loss of 23.6% in Pingyang Tezao. These results indicated that the disappearance of carboxyl groups caused reductions of 23.6%–40.0% in the F contents in the cell walls bound to carboxyl groups; therefore, 23.6%–40.0% of the F in the cell wall is closely associated with carboxyl groups, and these groups play an important role in binding F to the cell wall.

**Table 3:** F loss in cell wall induced by esterification modification.

Cultivars	Treatments	F content (mg/kg)	F loss amount (%)	Fluorine loss caused by esterification modification (%)
NKZ	Cell wall	7117.3 ± 101.4a		40.0
	Esterification	1713.6 ± 52.4c	75.9	
	CK	4559.0 ± 24.9b	35.9	
PYTZ	Cell wall	2270.4 ± 70.4a		23.6
	Esterification	268.4 ± 33.0c	88.2	
	CK	802.7 ± 25.5b	64.6	

Different lowercase letters indicated significant differences between the same cultivar (*p*<0.05.).

**Figure 3:**
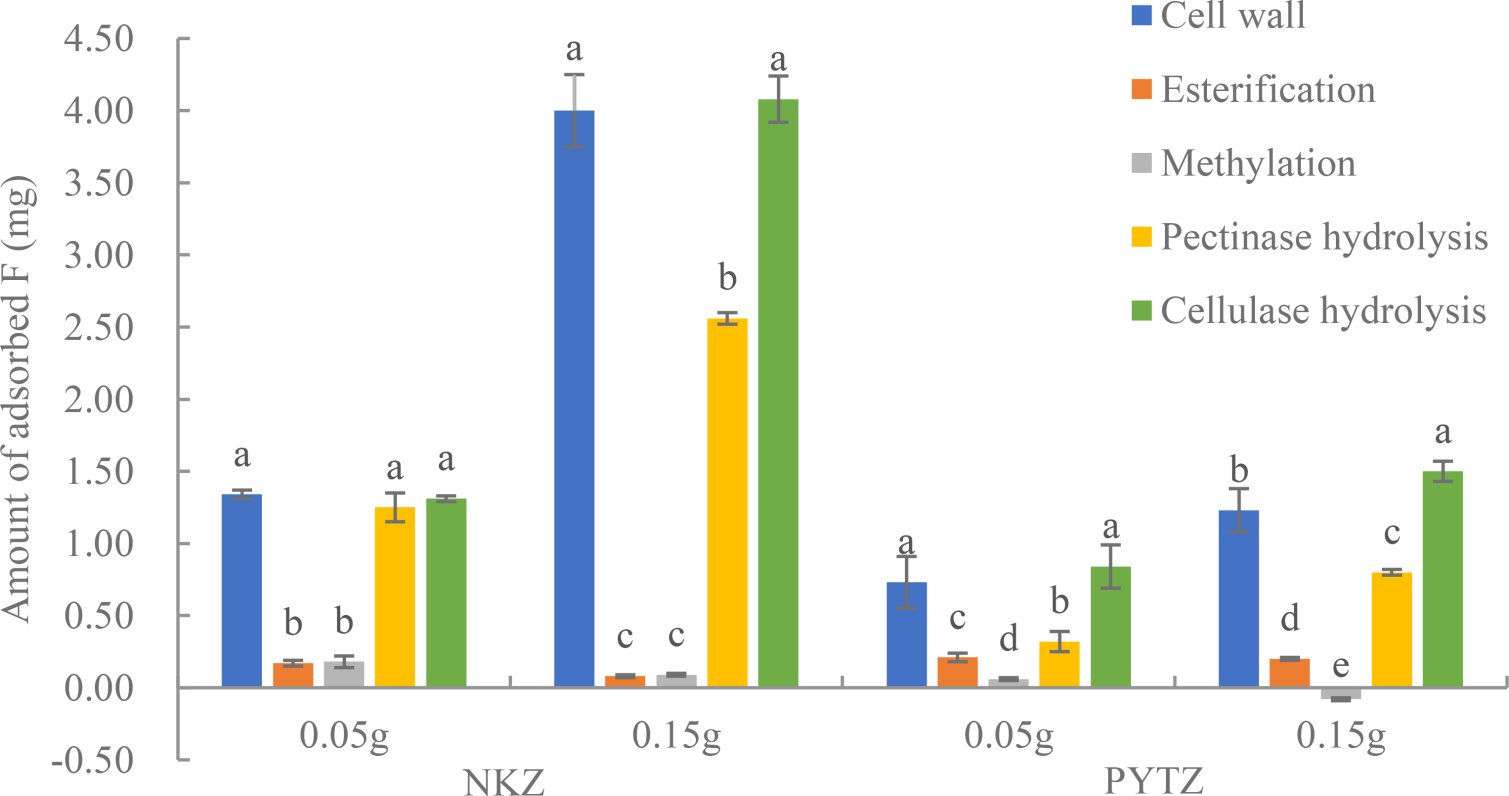
F adsorption by NKZ and PYTZ cell wall before and after modification at different masses. Different lowercase letters in the same cultivar and quality indicate significant differences at *p*<0.05.

After the pectinase treatment, the pectin in the cell walls was hydrolyzed into β-galacturonic acid. **Table 4** indicated that, in Nongkangzao, the F contents in the cell wall before and after pectinase treatment were 7117.3 mg/kg and 4169.7 mg/kg, respectively, representing an F loss of 41.4%. In the control group, the F content decreased from 7117.3 mg/kg to 4151.7 mg/kg, with an F loss of 41.7%. The F loss caused by pectinase treatment did not differ significantly from the control group. In Pingyang Tezao, the F contents in the cell wall before and after pectinase treatment were 2270.3 mg/kg and 761.3 mg/kg, respectively, resulting in an F loss of 66.5%. In the control group, the F content decreased from 2270.3 mg/kg to 766.4 mg/kg, with an F loss of 66.0%. Similarly, the F loss due to pectinase modification did not differ significantly from the control group. Based on these observations, two possibilities can be proposed: (1) Pectinase treatment has minimal effect on the F content in the cell wall, meaning that the size of the pectin molecular chain does not influence F binding; (2) The size of the pectin molecular chain does influence F binding, but the F bound in pectin is more prone to decomposition and loss in water. Given the results of the F adsorption study of pectinase-modified cell walls (**Figure 3**), the second hypothesis appears to be more reasonable.

**Table 4:** F loss in cell wall induced by pectinase hydrolysis.

Cultivars	Treatments	F content (mg/kg)	F loss amount (%)	F loss induced by pectinase hydrolysis (%)
NKZ	Cell wall	7117.3 ± 101.4a		
	Pectinase hydrolysis	4169.7 ± 35.2b	41.4	
	CK	4151.7 ± 55.6b	41.7	-0.3
PYTZ	Cell wall	2270.4 ± 70.4a		
	Pectinase hydrolysis	761.3 ± 6.4b	66.5	
	CK	766.4 ± 19.9b	66.2	-0.3

Different lowercase letters indicated significant differences between the same cultivar (*p*<0.05.).

The effect of cellulase hydrolysis on the F content in tea plant leaf cell walls is presented in **Table 5**. The results indicated that for Nongkangzao, the F contents in the cell wall before and after cellulase hydrolysis were 7117.3 mg/kg and 4640.3 mg/kg, respectively, representing an F loss of 34.8%. In the control group, the F content decreased from 7117.3 mg/kg to 4683.3 mg/kg, with an F loss of 34.2%. The F loss induced by cellulase hydrolysis did not differ significantly from that in the control group. For Pingyang Tezao, the F contents in the cell wall before and after cellulase hydrolysis were 2270.3 mg/kg and 915.2 mg/kg, respectively, resulting in an F loss of 59.7%. In the control group, the F content decreased from 2270.3 mg/kg to 923.1 mg/kg, with an F loss of 59.3%. Again, the F loss due to cellulase hydrolysis did not differ significantly from the control group. Furthermore, considering that there was no significant difference in the F adsorption capacity between the cellulase-treated and untreated cell walls (**Figure 3**), it can be concluded that cellulase hydrolysis has minimal effect on the F content in the cell wall.

**Table 5:** F loss in cell wall induced by cellulase hydrolysis.

Cultivars	Treatments	F content (mg/kg)	F loss amount (%)	F loss induced by cellulase hydrolysis (%)
NKZ	Cell wall	7117.3 ± 101.4a		
	Cellulase hydrolysis	4640.3 ± 51.7b	34.8	
	CK	4683.3 ± 12.5b	34.2	0.6
PYTZ	Cell wall	2270.4 ± 70.4a		
	Cellulase hydrolysis	915.2 ± 57.6b	59.7	
	CK	923.1 ± 32.0b	59.3	0.5

Different lowercase letters indicated significant differences between the same cultivar (*p*<0.05).

### 3.5 Effect of modified cell wall on F adsorption

The adsorption of F by cell walls with different modifications before and after treatment is shown in **Figure 3**. The results indicated that F adsorption by 0.05 g esterified and methylated Nongkangzao cell walls decreased by 87.3% and 86.6%, respectively, compared with unmodified cell walls. For 0.05 g esterified and methylated Pingyang Tezao cell walls, F adsorption decreased by 71.2% and 91.8%, respectively, compared with the unmodified cell walls. When the mass was increased to 0.15 g, the F adsorption by esterified and methylated Nongkangzao cell walls decreased by 98.0% and 97.8%, respectively, compared with unmodified cell walls. For 0.15 g esterified Pingyang Tezao cell walls, the F adsorption decreased by 83.7%, and the methylated cell walls released F during the adsorption process. These results suggested that the esterified carboxyl group and the methylated amine group are the main functional groups involved in F binding. Furthermore, after pectinase hydrolysis, Nongkangzao and Pingyang Tezao cell walls showed a significant reduction in F adsorption, indicating that the size of the pectin molecular chains affects F binding. In contrast, the F adsorption by cell walls of both varieties did not differ significantly after cellulase hydrolysis, implying that cellulase hydrolysis has minimal effect on the binding between the cell wall and F.

## 4 Discussion

### 4.1 F content and speciation in the cell wall

The plant cell wall, as the first line of defense against external environmental stresses and adverse conditions, plays a crucial role in a plant’s resistance to abiotic stress (Malinovsky et al., 2014). The results of this study showed that F content in the cell walls of Nongkangzao and Pingyang Tezao tea leaves accounts for 83.3% and 80.8% of the total F in the fresh leaves, respectively, indicating that F is predominantly concentrated in the cell walls of tea leaves. Luo et al. (2021) analyzed the F content in the cell wall of the Echa 10 tea cultivar, revealing that F in the cell wall comprised 67%–92% of the total F content in the leaf. Fang (2014) analyzed the F content in the cell walls of the Wuniuzao, Fudingdabai, and Fuyun No. 6 tea varieties, finding that the proportions of F in the cell walls relative to total leaf F were 38.21%, 40.17%, and 45.74%, respectively. These proportions are notably smaller than the results of our study and Luo et al. (2021), which can be attributed to differences in the experimental methods. Fang used dialysis in their experiment, which washes out approximately 18%–28% of the F content, yielding a result closer to these findings. Other studies have shown that when tea seedlings are exposed to F at varying concentrations, the F content in the cell walls increases proportionally with the F concentration (Du, 2019). These findings further support the conclusion that F is primarily fixed in the cell walls of tea leaves. The cell wall prevents the further penetration of F into the internal cellular structures, effectively reducing F-induced damage to the cell organelles. This is a key reason why tea plants exhibit F tolerance.

Based on the experimental results, F in the tea plant cell wall is primarily present in a water-soluble form. In the high-F cultivar, Nongkangzao, water-soluble F accounted for 68.4% of the total F in the cell wall, while in the low-F cultivar, Pingyang Tezao, it accounted for 42.5%. Hu et al. (2019) analyzed the F speciation in the cell walls of three tea varieties and found that water-soluble F accounted for over 82% of the total F content in the cell walls of all three varieties; therefore, F is mainly fixed in the cell wall in a water-soluble form in tea plants.

### 4.2 The relationship between F and metal elements in the cell wall and its distribution in cell wall Polysaccharide components

The plant cell wall is primarily composed of polysaccharides such as pectin, cellulose, and hemicellulose. The experimental results presented in this study show that the F content in the pectin fraction of Nongkangzao and Pingyang Tezao tea leaves accounts for 83.2% and 89.6% of the total F content in the cell wall, respectively; therefore, pectin is the main component responsible for F accumulation in the cell wall. Luo et al. (2021), who treated Echa 10 tea seedlings with different F concentrations, also found that pectin was the primary component in the cell wall responsible for F accumulation, with the F content in pectin representing 56%–71% of the total F in the cell wall. Moreover, as the F concentration increased, the F content in the cell wall components such as pectin, cellulose, and hemicellulose showed a significant increase. Specifically, the F contents in pectin increased by 34%, 111%, 188%, and 242% at varying F concentrations compared with the control, with these differences being highly significant. Additionally, the F content in pectin was strongly correlated with the F concentration, and the pectin content in the cell wall also increased significantly with higher F concentrations (Du, 2019; Luo et al., 2021). This could explain why pectin is the primary component for F accumulation in the cell wall. Plant cell wall pectin is a type of polysaccharide mainly composed of galacturonic acid, which contains numerous negatively charged carboxyl groups, providing the primary binding sites for metal cations in the cell wall (Yan et al., 2022; Coculo and Lionetti, 2022). Al in peas and Mn in sugarcane are both found to accumulate in cell wall pectin (Yang et al., 2016, 2019). Cai et al. (2014) used X-ray photoelectron spectroscopy surface analysis to find that F in tea leaves is likely present in the form of complexes with metal ions, such as MgF_2_ and CaF_2_. In this study, a correlation analysis between F and metal elements in various cell wall components showed significant correlations between F and metals such as Al, Ca, K, and Mn (*p*<0.05). Wen’s results also indicate that fluoride in tea plant is significantly correlated with Al, Ca, and Mn (Wen et al, 2024). Hu et al. (2019) found similar results, demonstrating a significant positive correlation between F and Al, Fe, and Mn in the cell wall and a significant negative correlation with Mg ions. Luo (2020), who applied different F concentrations to tea seedlings, performed a correlation analysis of the F content in the cell wall and the contents of various elements in the cell wall. It found that the total F in the cell wall, F in pectin, and F in cellulose were all significantly positively correlated with Cu and Mg. F in pectin and hemicellulose showed a significant positive correlation with Zn. In contrast, the total F content in the cell wall and F content in its components showed a significant negative correlation with Fe (Luo, 2020). Although the results from Luo (2020) regarding metal elements correlated with F differ from those in this study, possibly due to differences in experimental approaches, both studies support the notion that F in the cell wall is closely related to metal elements.

Based on these findings, it can be inferred that in tea plants, F induces an increase in cell wall pectin content, thus providing more binding sites for metal ions. This, in turn, facilitates the accumulation of these ions in the tea leaf cell wall and the subsequent chelation of F within the cell wall, which helps protect internal cellular organelles from F toxicity. Luo’s research showed that F significantly increased the content of most metal elements and boron in the cell wall of tea leaves while also promoting the synthesis of cellulose and pectin polysaccharides (Luo, 2020). These findings further support our hypothesis regarding the role of pectin in F accumulation and protection within the cell wall.

### 4.3 F binding sites in the cell wall

The results of the cell wall modification experiments in this study indicate that F primarily binds to the carboxyl and amino functional groups in the cell wall. After modification of the cell wall via methylation, the abundant amino groups in the tea plant leaf cell wall were methylated and disappeared. As a result, the F contents in the cell walls of Nongkangzao and Pingyang Tezao decreased by 38.5% and 29.4%, respectively, due to the loss of amino groups, suggesting that amino groups play an important role in F binding in cell walls. The F adsorption experiments before and after methylation modification of the cell wall also support this conclusion. The F adsorption capacity of the methylated cell wall was significantly lower than that of the unmodified cell wall (**Figure 3**), indicating that after methylation, the reduction in amino groups led to fewer binding sites for F, thus decreasing the amount of F adsorbed. After modification of the cell wall via esterification, the carboxyl groups in the cell wall were removed.

The results of this study show that the F contents in the cell walls of Nongkangzao and Pingyang Tezao decreased by 40.0% and 23.6%, respectively, due to the esterification of carboxyl groups, suggesting that carboxyl groups play an important role in F binding in the cell wall. The F adsorption capacity of the esterified cell wall was significantly lower than that of the unmodified cell wall (**Figure 3**), further supporting the conclusion that F binds to carboxyl groups; therefore, F primarily binds to the amino and carboxyl groups in the cell wall, likely forming ionic or coordination bonds with metal ions, which are then fixed in the cell wall. Liu et al. (2018) found that after methylation and esterification of the cell wall, the F contents were reduced significantly, decreasing by 55.82% and 27.54%, respectively. After treatment with cellulose and pectinases, the changes in F content did not differ significantly from the control, suggesting that F mainly binds to the amino and carboxyl groups in the cell wall, consistent with the findings in this study. Luo (2020), using Fourier transform infrared spectroscopy, analyzed the changes in functional groups in the tea plant leaf cell wall under different F concentrations and found that F promoted the carboxyl group content in pectin, further confirming the conclusions obtained in this study. Therefore, tea plant cell protoplasts may avoid the toxicity of F due to the presence of a large amount of -NH_2_, -COOH in the cell wall pectin to bind F in the form of hydrogen bonds or the presence of a large number of metal ions to bind F and immobilize it in the cell wall.

## 5 Conclusion

In conclusion, F predominantly exists in a water-soluble form in the leaf cell wall. Further analysis of the F content in the cell wall components revealed that most F accumulates in the pectin fraction of the cell wall. F primarily binds to the amino and carboxyl groups in pectin, or form ionic or coordination bonds with metal ions, thereby being fixed in the cell wall, preventing F from entering the cell interior and mitigating its harmful effects on cellular organelles. This mechanism is crucial to the F tolerance observed in tea plants.

## Data Availability

The original contributions presented in the study are included in the article/supplementary material. Further inquiries can be directed to the corresponding author.

## References

[B1] Cai H. M. Dong Y. Y. Li Y. Y. Peng D. X. Zhang C. Y. Wan X. X. C.. (2016). Physiological and cellular responses to fluoride stress in tea (*Camellia sinensis*) leaves. Acta Physiol. Plant 38, 144–155. doi: 10.1007/s11738-016-2156-0

[B2] Cai H. Peng C. Chen J. Hou R. Gao H. Wan X. (2014). X-ray photoelectron spectroscopy surface analysis of fluoride stress in tea (*Camellia sinensis* (L.) O. Kuntze) leaves. J. Fluor. Chem. 158, 11–15. doi: 10.1016/j.jfluchem.2013.11.012

[B3] Cai H. M. Peng C. Y. Li C. L. Gao Z. Hou R. Y. Wan X. C. (2013). Fluoride accumulation and its subcellular distribution in three tea plants. Scientia Agricultura Sin. 46, 1668–1675. doi: 10.3864/j.issn.0578-1752.2013.08.016

[B4] Coculo D. Lionetti V. (2022). The plant invertase/pectin methylesterase inhibitor superfamily. Front. Plant Sci. 13. doi: 10.3389/fpls.2022.863892 PMC8990755 35401607

[B5] Du Y. R. (2019). Studies on Mechanism of Cell Wall Pectin and Hemicellulose Involved in the Fluorine Accumulation in Tea Leaves (Wuhan: Huazhong Agricultural University).

[B6] Fang F. X. (2014). Chemical form, location and enrichment mechanism of fluorine in tea leaves (Wuhan: Huazhong Agricultural University).

[B7] GB5009.268-2016 (2016). National Standards for Food Safety: Determination of Multiple Elements in Foods (Beijing, CN: China Food and Drug Administration).

[B8] Hu N. Fang F. X. Du Y. R. Chen Y. Q. (2019). Subcellular distribution and chemical forms of fluoride in tea tree leaves (*Camellia sinensis* L.) and its cell walls. Fluoride 52, 385–396. Available online at: https://www.fluorideresearch.org/epub/files/027.pdf.

[B9] Li C. L. (2011). Study on the effect and mechanism of fluoride in the physiology and biochemistry of tea seedlings (Wuhan: Huazhong Agricultural University).

[B10] Li C. Z. Tao J. Zhao D. Q. You C. Ge J. T. (2012). Effect of calcium sprays on mechanical strength and cell wall fractions of herbaceous peony (Paeonia Lactiflora pall.) inflorescence stems. Int. J. Mol. Sci. 13, 4704–4713. doi: 10.3390/ijms13044704 22606005 PMC3344241

[B11] Li C. L. Zheng Y. N. Zhou J. R. Xu J. Ni D. J. (2011). Changes of leaf antioxidant system, photosynthesis and ultrastructure in tea plant under the stress of fluorine. Biol. Plantarum. 55, 563–566. doi: 10.1007/s10535-011-0126-3

[B12] Liu S. Y. Zhu X. J. Fang F. X. H.j. Z. Qiu A. D. Chen Y. Q. (2018). Fluorine Subcellular distribution and its combining characteristics with cell wall in tea leaves (*Camellia sinensis*). J. Tea Sci. 38, 305~312. doi: 10.13305/j.cnki.jts.2018.03.011

[B13] Luo J. L. (2020). Study on Mechanism of Fluorine Accumulation in Cell Wall of Tea Plant Leaf (Wuhan: Huazhong Agricultural University).

[B14] Luo J. L. Ni D. J. Li C. L. Du Y. R. Chen Y. Q. (2021). The relationship between fluoride accumulation in tea plant and changes in leaf cell wall structure and composition under different fluoride conditions. Environ. pollut. 270, 116283. doi: 10.1016/j.envpol.2020.116283 33341550

[B15] Malinovsky F. Fangel J. Willats W. (2014). The role of the cell wall in plant immol/Lunity. Front. Plant Science. 5. doi: 10.3389/fpls.2014.00178 PMC4018530 24834069

[B16] Pan J. T. Li D. Q. Zhu J. J. Shu Z. F. Ye X. L. Xing A. Q.. (2020). Aluminum relieves fluoride stress through stimulation of organic acid production in *Camellia sinensis* . Physiol. Mol. Biol. Plants 26, 1127–1137. doi: 10.1007/s12298-020-00813-2 32549678 PMC7266864

[B17] Shu W. S. Zhang Z. Q. (2003). Fluoride and aluminium concentrations of tea plants and tea products from Sichuan Province, PR China. Chemosphere 52, 1475–1482. doi: 10.1016/S0045-6535(03)00485-5 12867178

[B18] Wang L. X. (2014). Fluoride Accumulation in tea plant and its physiological response mechanism (Yanglin: Northwest A&F University).

[B19] Wen X. J. Wang Y. C. Wang S. T. Yao N. Wu X. M. Fawad Z.. (2024). Fawad Zaman Fluorine accumulation characteristics of 85 tea tree (*Camellia sinensis*) varieties and its potential risk assessment. Ecotox Environ. Safe 283, 116785. doi: 10.1016/j.ecoenv.2024.116785 39067075

[B20] Wu W. H. Xie Z. M. Xu J. M. Hong Z. P. Liu C. Wu W.. (2002). Characteristics of forms of fluorine in soils and influential factors. Chin. J. Enviromental Sci. 23, 104–108. doi: 10.3321/j.issn:0250-3301.2002.02.022 12048804

[B21] Xu J. (2011). Mechanisms of lead uptake/accumulation and tolerance in tea plant (Camellia sinensis) (Hangzhou: Zhejiang University).

[B22] Yan L. Li S. Cheng J. Liu Y. Liu J. Jiang C. (2022). Boron contributes to excessive aluminum tolerance in trifoliate orange (Poncirus trifoliata (L.) Raf.) by inhibiting cell wall deposition and promoting vacuole compartmentation. J. Hazard. Mater. 437, 129275. doi: 10.1016/j.jhazmat.2022.129275 35714543

[B23] Yang J. Mei Q. Jing F. Fang S. R. Ming F. Y. You L. J.. (2016). Alkali-soluble pectin is the primary target of aluminum immobilization in root border cells of Pea (Pisum sativum). Front. Plant Sci. 7. doi: 10.3389/fpls.2016.01297 PMC5020075 27679639

[B24] Yang S. Yi K. Chang M. M. Ling G. Z. Zhao Z. K. Li X. F. (2019). Sequestration of Mn into the cell wall contributes to Mn tolerance in sugarcane (Saccharum officinarum L.). Plant Soil 436, 475–487. doi: 10.1007/s11104-019-03937-x

[B25] Yang Y. H. Yu M. Du C. W. Wu S. L. Wang Y. H. (2005). Boron distribution in cell wall components in rape cultivars (Brassica napus)with different boron use efficiency. Acta Agronomica Sin. 31, 608–611. doi: 10.3321/j.issn:0496-3490.2005.05.014

[B26] Yang X. Yu Z. Zhang B. B. Huang J. Zhang Y. H. Fang F. X.. (2015). Effect of fluoride on the biosynthesis of catechins in tea [*Camellia sinensis* (L.) O. Kuntze] leaves. Scientia Horticulturae. 184, 78–84. doi: 10.1016/j.scienta.2014.12.031

