# Supplementary material for: Research on the distribution, localization, and morphology of fluorides in the cell walls of tea plant leaves
Source: Front Plant Sci. 2025 Mar 6;16:1539883. doi: 10.3389/fpls.2025.1539883 (PMC11923811; doi:10.3389/fpls.2025.1539883)
Supplement: Supplementary file 1 [file Table1.docx]

TABLE Analysis of metal element content in Nongkangzao cell wall components (mg kg^-1^)

| Elements | Cell wall components | | | |
| --- | --- | --- | --- | --- |
|  | Chelated pectin | Alkali soluble pectin | Hemicellulose | Cellulose |
| Al | 4553.4±158.2a | 758.2±29.9c | 758.4±12.0c | 2758.1±157.7b |
| Ca | 1098.2±58.6c | 11100.2±190.5b | 270.6±20.6d | 14440.3±241.0a |
| Cu | 35.6±1.58a | 17.1±0.9b | 3.9±0.2d | 7.2±0.6c |
| Fe | 242.2±13.5b | 74.1±5.2c | 22.6±3.7d | 427.3±20.1a |
| K | 18550.0±141.8c | 7710.3±105.6d | 26630.6±352.2b | 35881.5±241.3a |
| Mg | 551.6±13.3c | 2184.2±39.5a | 19.7±1.1d | 782.6±14.1b |
| Mn | 713.5±26.8c | 10310.1±105.8a | 31.9±1.6d | 1546.3±15.4b |

Different small letters in the same row mean significant differences at *p*<0.05.

TABLE Analysis of metal element content in the cell wall components of Pingyang Tezao (mg kg^-1^)

| Elements | Cell wall components | | | |
| --- | --- | --- | --- | --- |
|  | Chelated pectin | Alkali soluble pectin | Hemicellulose | Cellulose |
| Al | 4868.4±151.8a | 975.9±30.4b | 238.2±20.0c | 1345.2±109.5d |
| Ca | 1378.3±23.4c | 9981.2±56.9a | 191.9±8.7d | 4420.4±48.4b |
| Cu | 26.2±2.3a | 16.1±1.2b | 3.0±0.2d | 5.3±0.4c |
| Fe | 291.5±7.6b | 74.4±5.5c | 34.1±1.8d | 413.3±20.4a |
| K | 20260.2±204.1c | 8692.3±89.5d | 26112.2±120.4b | 29571.6±185.2a |
| Mg | 469.4±18.3c | 3450.0±59.2a | 28.1±2.5d | 644.5±22.3b |
| Mn | 632.3±15.7c | 5881.3±105.3a | 23.6±2.3d | 849.7±33.5b |

Different small letters in the same row mean significant differences at *p*<0.05.

TABLE Correlation analysis of fluorine and metal elements in cell wall fractions

| elements | F | Al | Ca | Cu | Fe | K | Mg | Mn |
| --- | --- | --- | --- | --- | --- | --- | --- | --- |
| Correlation coefficient | 1.0000 | 0.7396* | 0.7252* | 0.4297 | 0.3542 | -0.7269* | 0.5354 | 0.7853* |

Note:* *p*<0.05
